# Maternal child maltreatment and trajectories of offspring behavioural and emotional difficulties from age 4 to 7 years – results from a prospective birth cohort study

**DOI:** 10.1007/s00787-024-02534-3

**Published:** 2024-07-22

**Authors:** Vera Clemens, Deborah Wernecke, Jörg M. Fegert, Jon Genuneit, Dietrich Rothenbacher, Stefanie Braig

**Affiliations:** 1 Department of Child and Adolescent Psychiatry/Psychotherapy, University Hospital Ulm, Ulm, Germany; 2 German Center for Mental Health (DZPG), Partner site Ulm, Ulm, Germany; 3 Institute of Epidemiology and Medical Biometry, Ulm University, Helmholtzstraße 22, 89081 Ulm, Germany; 4 German Center for Children and Youth Health, Partner site Ulm, Ulm, Germany; 5 Pediatric Epidemiology, Department of Pediatrics, Medical Faculty, Leipzig University, Leipzig, Germany

**Keywords:** Maternal experience of child maltreatment, Strength and Difficulties Questionnaire (SDQ), Trajectories, Course of child mental health

## Abstract

Maternal experience of child maltreatment (CM) has been associated with maternal and child mental health. However, evidence about the course of child mental health and maternal CM is scarce. Therefore, this study aims to compare trajectories of mental health in children according to maternal CM exposure and maternal mental health. We included 327 mothers and their singleton child from the Ulm SPATZ Health Study, a prospective birth cohort study. Child mental health was determined by the Strength and Difficulties Questionnaire (SDQ) at the age of 4, 5, 6, and 7 years and maternal CM by the Childhood Trauma Questionnaire (CTQ). Results display that maternal CM is associated with more behavioural and emotional difficulties in children, a trend that tends to increase with older child age. The sum of maternal mental health problems across this time course mediates this association. Male child sex is associated with more mental health problems in the child and the mother. These results provide an important first insight into the relevance of maternal CM on the trajectories of mental health in the offspring and highlight the importance of chronicity and severity of maternal mental health. Further prospective research in cohorts with longer follow-ups up into adolescence and adulthood is needed.

**Supplementary Information:**

The online version contains supplementary material available at 10.1007/s00787-024-02534-3.

## Introduction

The experience of child maltreatment (CM) can impair later life substantially. Sequelae include mental and physical diseases, psychosocial and economic problems, a decreased quality of life [1–4], and a reduced life expectancy of up to 20 years [5]. CM, defined as ‘any act or series of acts of commission or omission by a parent or other caregiver that results in harm, potential for harm, or threat of harm to a child’ [6], encompasses emotional, physical and sexual abuse, as well as emotional and physical neglect. CM is estimated to be experienced by 8–35% of children/adolescents globally [7]. Thus, CM can be considered a major public health problem and one of the most important preventable risk factors for health [8].

Research suggests that the risks of CM go beyond one lifespan. Maternal CM exposure was shown to be associated with a higher prevalence of externalizing and internalizing problems in the mother`s own children across childhood and adolescence [9, 10]. Possible reasons for this intergenerational transmission of the long-term effects of CM can be pre- and postnatal. Prenatal transmission may involve alterations in the maternal oocytes, e.g. via epigenetic modifications or due to gestational biology, thus adjustments of the fetus to CM-related changes in the pregnant mother. Postnatal transmission may be linked to maternal CM-associated stress, mental health problems, own CM practices, and suboptimal or harsh parenting [11].

Several studies have shown the relevance of parental mental health in the interplay between parental CM and child mental health [12]. Parental mental illness is an established risk factor for child mental health, with more than 40% of children of mentally ill parents experiencing clinically evidenced or possible mental health problems [13]. Interestingly, while the effect of child sex, socio-economic status, maternal hostility, and child temperament on child mental health largely persist over time, the effects of maternal mental health increase slightly [14]. Literature shows that most parental psychiatric disorders result in non-specific child psychiatric disorders [15]. Exemplarily, the depression of a mother does not necessarily result in a depressive disorder in the child. Instead, other non-specific behavioral problems often occur. Importantly, health consequences of maternal depressive disorders are reversible, as effective treatment of maternal depression is associated with a significant decrease in child psychiatric symptoms [16].

An important moderator of the association between maternal CM and child mental health might be child (birth) sex. For instance, findings revealed that child sex moderates the relation between sexual trauma history and maternal behaviour towards children [17]. According to Letourneau and colleagues, sex differences in the intergenerational transmission of CM via maternal anxiety and depression were observed, with boys showing greater vulnerability to the indirect effects of maternal CM via anxiety and depression [18]. Male sex is associated with higher behavioural and emotional difficulties compared to female peers, and furthermore with a less strong decrease of higher behavioural and emotional difficulties over age. While in a representative sample of children in Germany, behavioural and emotional difficulties measured using the Strength and Difficulties Questionnaire (SDQ) [19], decreased by 1.43 points between the age of 4 to 7 years, the reduction in male peers consisted of only 0.16 points [20].

Together, literature points towards an intergenerational effect of maternal CM on mental health of the offspring with maternal mental health and child sex affecting this interplay. However, previously published longitudinal data in children are sparse and lack detailed characterization of maternal CM and child mental health trajectories. From a developmental perspective, the influence of maternal CM in the offspring may increase over time in a cascadian way by influencing more and more factors, such as maternal mental health [21].

Firstly (1), we aimed to show the trajectories of child behavioural and emotional difficulties across maternal CM exposure in children aged between 4 and 7 years. Additionally (2), we depicted trajectories of maternal mental health across maternal CM exposure in the same time frame. Moreover (3), we conducted a path analysis to examine the relationship between maternal CM and child behavioural and emotional difficulties, mediated by maternal mental health. Further goals of our study were to explore (4) the role of child sex in the above mentioned associations/trajectories and (5) to understand the time-dependent effect, i.e. potential changes in the relationship over child age.

## Methods

### Study design and study population

We analyzed data from a prospective birth cohort recruited from the general population, the Ulm SPATZ Health Study. All women who came to the University Medical Centre Ulm, Germany, which was the only maternity hospital in Ulm during the recruitment period between 04/2012 and 05/2013 for the delivery of their baby were asked to participate. The overall response rate was 49%, resulting in *n* = 1,006 mothers with 934 singleton children at baseline. Participation was voluntary and informed consent was obtained for each participant (details in [22]). The study was approved by the ethics board of Ulm University (no. 311/11). Postal follow-up questionnaires for the children, the mothers, and their partners were sent to the families on an annual basis, including at ages 4, 5, 6, and 7 years of the child’s life, which are relevant for this study. The selection of the age range was based on data availability and on the age range of the questionnaires used (please see the following paragraph for the outcome measure).

### Outcome measure

The Strength and Difficulties Questionnaire (SDQ, in its validated German version) [19] was completed annually by the parents, mostly the mothers. The SDQ is widely used and measures emotional and conduct problems, hyperactivity, peer relationship problems, and prosocial behaviour. Missing values were replaced by the mean of the remaining variables of the subscale if number of missing values was ≤ 2 (*n* = 6 for conduct problems at age 6 years, less often for other subscales and other time points). Internal consistency ranged from 0.72 (emotional problems at age 4 years) to 0.80 (hyperactivity at age 7 years). The total difficulties score is composed of the scores of all subscales but prosocial behaviour and ranges from 0 to 40, with higher scores suggesting greater problem behaviour.

### Exposure measure

The Childhood Trauma Questionnaire (CTQ), short form [23, 24] was part of the postal follow-up at child age 7 years to identify the extent of maternal CM. The CTQ consists of five dimensions: emotional abuse, physical abuse, sexual abuse, emotional neglect, and physical neglect, each containing five items. The items for trivialization were not considered. The total value of each subscale ranges from five points (no CM) up to 25 points (extreme experiences of CM). Cronbach’s alpha ranged from 0.73 (physical neglect) to 0.83 (sexual abuse). We dichotomized the variables (0 = not or minimal versus 1 = more than minimal) according to Häuser et al. 2011 [25] and summed these variables up resulting in the “sum of CM forms” (0, 1, ≥ 2). Three participants had two missing values on different scales. Eight participants had one missing value. No substitution of missing values was done for the exposure measure. We have chosen to focus on the different forms of CM as diverse effects of e.g. sexual abuse compared to neglect are described [26, 27] instead of using a simplistic approach by collapsing items in one total score thus losing a lot of important information [28]. However, as different types of CM are highly interrelated [29] and the cumulative effect of CM types is well known [30] we have decided to use both approaches, focusing on individual forms of CM and a summary score of experienced CM forms.

### Covariates

Maternal age at childbirth (≤ 25, 26–35, ≥ 36 years) and child (biological) sex at birth was derived from the clinic information system. Sociodemographic, familial characteristics (including number of persons living in the same household with the child), and lifestyle factors were assessed at baseline by a self-administered standardized questionnaire handed out to the mother shortly after delivery: Maternal nationality (derived from nationality and country of birth), maternal education (< 12 years, ≥ 12 years of school education), maternal smoking in the year before pregnancy (yes, no). Covariates were chosen following previous literature, e.g. [31, 32] and depending on data availability.

The German version of HADS (Hospital Anxiety and Depression Scale) [33], referring to symptoms of anxiety and depression was completed annually by the mothers concerning the week prior to the follow-up assessment. The summary score of each subscale ranges from 0 to 21. For each subscale, missing values were replaced by the mean of the remaining items of the same scale if there was not more than one missing value (*n* = 1 for depression symptoms at age 4, *n* = 2 for depression symptoms at age 5). Anxiety disorders and depression comprise the most frequent mental health disorders in Germany [34]. As they were highly interrelated [34] (Spearman correlations of ≥ 0.58 in our analyses), scores of anxiety and depression symptoms were collapsed into one variable. A summary score of both subscales as a reliable indicator for various mental disorders is also possible according to the evaluation instructions.

### Statistical analysis

**Fig. 1:**
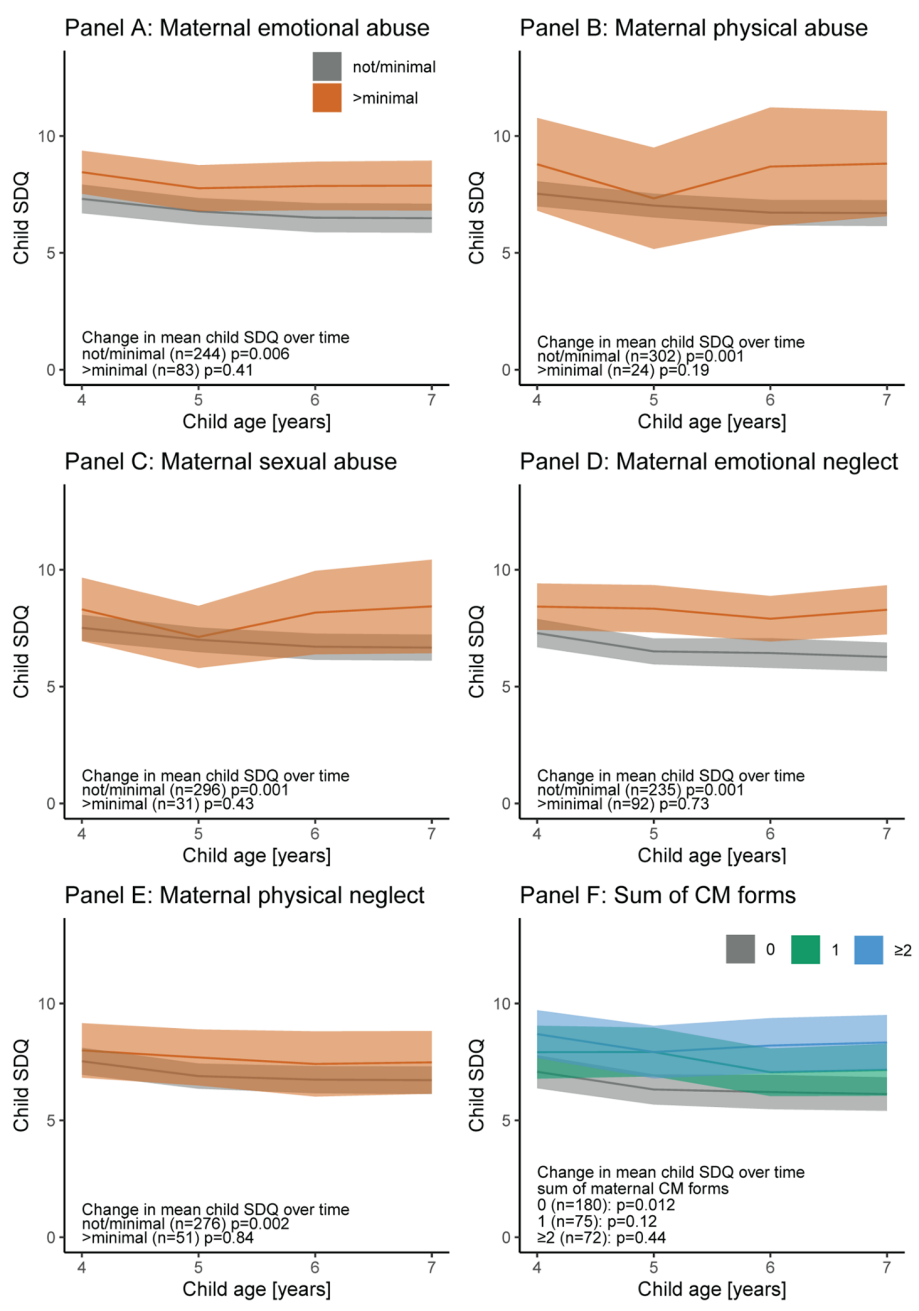
Mean predicted child SDQ and 95% confidence intervals according to maternal child maltreatment (adjusted for child sex). SDQ: Child behaviour difficulties

We show descriptive analyses and used ANOVAs and Chi-square tests (Table 1) to analyze sex differences in exposure, mediating or outcome variables (please see aim 4 specified in the introduction). We depict mean child SDQ trajectories adjusted for child sex (LSMEANS) according to maternal CM exposure including 95% confidence intervals (Fig. 1, aim 1). LSMEANS were derived from mixed models with child age as random effect, maternal CM, and sex as fixed effect. Changes over time were tested using the Type III p value. Similarly, we depict the trajectories of anxiety/depression symptoms according to maternal CM (see Online Resource, aim 2). Path analyses between maternal CM and child behaviour symptoms (SDQ) at age 7 years were conducted given a theoretical model including anxiety/depression symptoms (HADS) as a mediating variable (aim 3). For path analyses, as opposed to mixed model analyses, the area under the curve was used for anxiety/depression symptoms summing up maternal symptoms from child age 4 to 6 years. For calculating the area under the curve, missing values were replaced by the 25th percentile of the remaining participants at the corresponding time point. We conducted further mixed model analyses using child age as random term and continuously measured CM experiences, child sex, and an interaction of maternal childhood trauma*child age (continuously measured) as fixed. We introduced an interaction term to test heterogeneous trends in the associations over child age (aim 5). Before running the mixed model analyses, we tested the covariance structure and finally used an unstructured covariance matrix due to best fit. In preliminary analysis, we checked whether the covariates were confounders, this means that they were statistically significantly associated with the exposure and the outcome. For path analyses and mixed models, continuous variables were centered at the grand-mean, which allows to interpret the intercept of the model. A comparison of sociodemographic variables between the study population and the baseline population is presented in the Online Resource, Table 1. The analyses were performed using SAS^®^ 9.4 (The SAS Institute, Cary, NC, USA) and R (R Foundation for Statistical Computing, Vienna, Austria). Graphs were created by using R.

## Results

The study population consisted of 327 mothers and their singleton child (Table 1). Most (93.9%) were of German nationality. Maternal education at the time of delivery was high with 72.0% of participants with ≥ 12 years of education. More than a quarter of the mothers (25.4% and 28.1%, respectively) had experienced more than minimal emotional abuse or emotional neglect. Physical abuse was more rare (7.4% > minimal). Child SDQ with a mean value of 7.5 (SD = 4.7) at age 4 decreased with growing child age.

### Preliminary analyses

Maternal education did not contribute to the models and did not qualify as confounder (based on Akaike information criterion and p value ≥ 0.05). Maternal education was thus not considered in the final analyses, so was maternal age at childbirth, maternal nationality, and smoking before pregnancy.

**Table 1:** Characteristics of the study population (mothers and stratified according to offspring sex) at baseline and follow-up (FU)

Study population				
	Total (*n* = 327) *n* (%)	Boys (*n* = 155) *n* (%)	Girls (*n* = 172) *n* (%)	*p* value (sex difference)
Maternal age at childbirth [years]				0.22
≤ 25	10/327 (3.1%)	6/155 (3.9%)	4/172 (2.3%)	-
26–35	230/327 (70.3%)	102/155 (65.8%)	128/172 (74.4%)	-
≥ 36	87/327 (26.6%)	47/155 (30.3%)	40/172 (23.3%)	-
Mean (SD)	33.5 (4.5)	33.6 (4.8)	33.4 (4.1)	0.70
Maternal nationality German	307/327 (93.9%)	144/155 (92.0%)	163/172 (94.8%)	0.48
Single motherhood at childbirth	4/326 (1.2%)	1/154 (0.7%)	3/172 (1.7%)	0.37
Maternal education (< 12 years)	91/325 (28.0%)	43/153 (28.1%)	48/172 (27.9%)	0.97
Maternal smoking before pregnancy	58/326 (17.8%)	29/155 (18.7%)	29/171 (17.0%)	0.68
Maternal anxiety and depression symptom score (HADS)				
at 4 year FU, Mean (SD)	9.1 (5.7)	9.5 (5.7)	8.8 (5.7)	0.27
at 5 year FU, Mean (SD)	8.9 (5.4)	9.6 (5.7)	8.3 (5.1)	0.04
at 6 year FU, Mean (SD)	9.7 (5.7)	10.6 (6.2)	8.9 (5.2)	0.01
at 7 year FU, Mean (SD)	nA	nA	nA	nA
Maternal CM				
Emotional abuse > minimal	83 (25.4%)	37 (23.9%)	46 (26.7%)	0.55
Physical abuse > minimal	24 (7.4%)	9 (5.8%)	15 (8.8%)	0.31
Sexual abuse > minimal	31 (9.5%)	17 (11.0%)	14 (8.1%)	0.38
Emotional neglect > minimal	92 (28.1%)	42 (27.1%)	50 (29.1%)	0.69
Physical neglect > minimal	51 (15.6%)	23 (14.8%)	28 (16.3%)	0.72
Sum of CM forms > minimal				0.32
0	180 (55.1%)	83 (53.6%)	97 (56.4%)	-
1	75 (22.9%)	41 (26.5%)	34 (19.8%)	-
≥ 2	72 (22.0%)	31 (20.0%)	41 (23.8%)	-
Child SDQ total				
at 4 years, Mean (SD)	7.5 (4.7)	8.1 (4.6)	7.0 (4.6)	0.03
at 5 years, Mean (SD)	7.1 (4.5)	8.0 (4.8)	6.2 (4.1)	< 0.001
at 6 years, Mean (SD)	6.9 (4.8)	7.6 (5.4)	6.2 (4.1)	0.02
at 7 years, Mean (SD)	6.8 (5.0)	7.6 (5.4)	6.1 (4.5)	0.01

CTQ: Childhood trauma questionnaire, FU: follow-up, HADS: Hospital Anxiety and Depression Scale, SD: standard deviation, SDQ: Child behaviour difficulties, nA: not applicable

### Child behavioural and emotional difficulties according to maternal CM exposure (aim 1)

Child SDQ was higher if mothers had experienced more than minimal CM compared to mothers who had not (Fig. 1). Mostly however, the differences did not reach statistical significance. Nonetheless, children whose mothers had experienced emotional neglect showed statistically higher SDQ at ages 5 and 7 years (Fig. 1, Panel D) and importantly, a statistically significant difference in SDQ was evident if child SDQ was compared in mothers with 0 versus ≥ 2 forms of CM (Fig. 1, Panel F). Additionally, mean child SDQ decreased statistically significantly over the time if mothers had experienced no or minimal CM (see Type III p values), but no change for the better in SDQ with age was shown in children of mothers who had experienced CM classified as > minimal.

### Maternal mental health (aim 2)

With growing child age, maternal anxiety/depression symptoms have worsened (Online Resource Fig. 1). Statistically significant higher maternal anxiety/depression symptoms were associated with experiences of maternal emotional abuse > minimal (at child age 5 years, Panel A), maternal sexual abuse > minimal (at child age 6 years, Panel C) and the summative index of CM (0 versus ≥ 2 forms of CM, Panel F).

### Path analyses (aim 3)

**Fig. 2:**
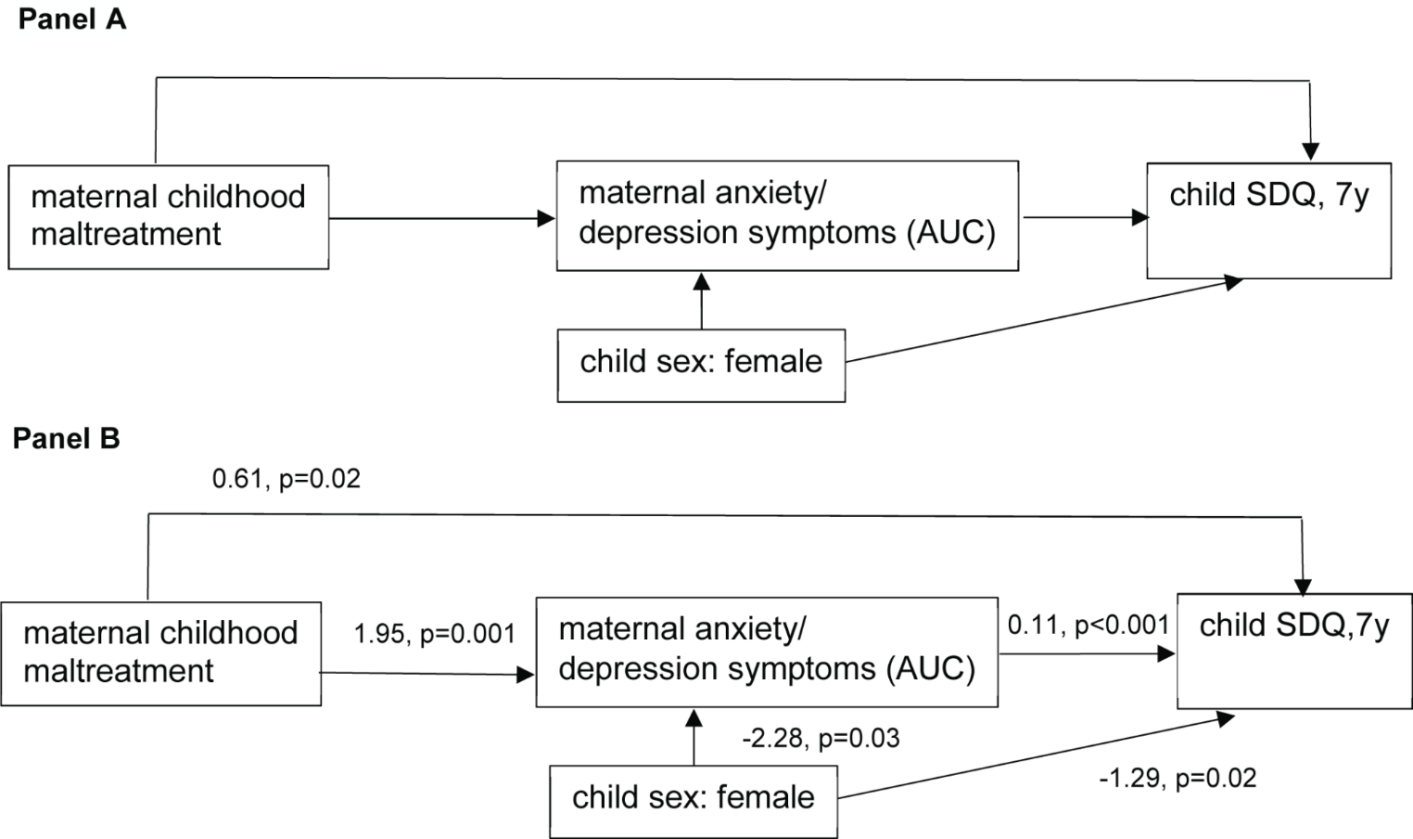
Panel A: Path analysis: Theoretical model. Panel B: Results: Sum of maternal childhood maltreatment forms (Akaike information criterion: 5741.73, Bayesian information criterion: 5779.54). AUC: Area under the curve, SDQ: Child behaviour difficulties, y: years

The theoretical model of the path analysis is shown in Fig. 2, Panel A including maternal anxiety/depression symptoms as mediating variable and child sex as a potential confounder. The sum of maternal CM forms was significantly associated with child SDQ at age 7 years, directly (coefficient (b) = 0.61, *p* = 0.02) and mediated by maternal anxiety/depression symptoms (b = 1.95, *p* = 0.001; b = 0.11, *p* < 0.001) (Fig. 2, Panel B). Similar results were shown for maternal childhood emotional abuse and maternal emotional neglect (Online Resource Fig. 2, Panel A, Panel D). We did not find an indirect path of maternal childhood physical abuse (Panel B) or sexual abuse (Panel C) on child SDQ via anxiety/depression symptoms. Interestingly, no direct path was seen for maternal childhood physical neglect on child SDQ (Panel E).

### The role of child sex (aim 4)

Male child sex was associated with increased symptoms of anxiety and depression in mothers and higher child SDQ compared to females (Table 1). Furthermore, path analyses including a possible interaction between maternal CM and child sex were performed. Interestingly, the highest interaction was shown in sexual abuse. However, the model fit was higher in the models without interactions than in those with interactions, which led us to neglect the interactions with sex in path analyses.

### Mixed model analyses (aim 5)

In mixed model analyses, maternal childhood emotional abuse was significantly associated with higher child SDQ (b: 0.17, *p* = 0.02) and decreasing SDQ with child age was shown (estimate (b): -0.58, -0.76, -0.77 at years 5, 6, 7 compared to 4) (Table 2). Similar associations between maternal CM and child SDQ were evident for emotional neglect (b: 0.24, *p* = 0.01) and the sum of CM experiences (b: 0.81, *p* = 0.01)). There was a statistically significant positive interaction between maternal sexual abuse and child age at 7 years of age. This suggests that the effect of maternal sexual abuse on the child SDQ increases with child age. For sexual abuse, physical abuse, and physical neglect, maternal CM was not statistically significant associated with child SDQ.

**Table 2:** Adjusted associations between maternal CM and chid SDQ. Results of mixed model analyses (adjustment was done for all variables mentioned in the specific section of the table)

Predictor		Estimate b	*p value*	Lower 95%CL	Upper 95%CL
Emotional abuse					
Intercept		**8.39**	**< 0.001**	7.70	9.07
Maternal emotional abuse (EMA)	cont.	**0.17**	**0.02**	0.03	0.31
Child age	4y	0			
	5y	**-0.58**	**0.01**	-1.05	-0.12
	6y	**-0.76**	**< 0.001**	-1.24	-0.28
	7y	**-0.77**	**< 0.001**	-1.24	-0.30
EMA*child age	4y	0			
	5y	-0.04	0.54	-0.17	0.09
	6y	0.11	0.10	-0.02	0.24
	7y	0.07	0.28	-0.06	0.20
Child sex	female	**-1.59**	**< 0.001**	-2.45	-0.71
	male	0			
Physical abuse					
Intercept		**8.37**	**< 0.001**	7.68	9.07
Maternal physical abuse (PHA)	cont.	0.15	0.30	-0.13	0.43
Child age	4y	0			
	5y	**-0.59**	**0.01**	-1.06	-0.13
	6y	**-0.73**	**< 0.001**	-1.22	-0.25
	7y	**-0.75**	**< 0.001**	-1.22	-0.29
PHA*child age	4y	0			
	5y	-0.17	0.20	-0.43	0.09
	6y	0.21	0.14	-0.07	0.48
	7y	0.20	0.13	-0.06	0.45
Child sex	female	**-1.55**	**< 0.001**	-2.43	-0.67
	male	0			
Sexual abuse					
Intercept		**8.35**	**< 0.001**	7.66	9.04
Maternal sexual abuse (SXA)	cont.	0.15	0.24	-0.10	0.40
Child age	4y	0			
	5y	**-0.58**	**0.01**	-1.04	-0.12
	6y	**-0.75**	**< 0.001**	-1.23	-0.27
	7y	**-0.77**	**< 0.001**	-1.23	-0.30
SXA*child age	4y	0			
	5y	-0.07	0.54	-0.28	0.15
	6y	0.12	0.30	-0.11	0.35
	7y	**0.22**	**0.04**	0.01	0.45
Child sex	female	**-1.50**	**< 0.001**	-2.37	-0.63
	male	0			
Emotional neglect					
Intercept		**8.35**	**< 0.001**	7.67	9.03
Maternal emotional neglect (EMN)	cont.	**0.24**	**0.01**	0.06	0.43
Child age	4y	0			
	5y	**-0.59**	**0.01**	-1.05	-0.12
	6y	**-0.75**	**< 0.001**	-1.21	-0.27
	7y	**-0.77**	**< 0.001**	-1.23	0.30
EMN*child age	4y	0			
	5y	0.07	0.43	-0.10	0.23
	6y	0.10	0.28	-0.08	0.27
	7y	0.10	0.23	-0.07	0.27
Child sex	female	**-1.49**	**< 0.001**	-2.34	-0.63
	male	0			
Physical neglect					
Intercept		**8.39**	**< 0.001**	7.69	9.08
Physical neglect (PHN)	cont.	0.08	0.56	-0.19	0.34
Child age	4y	0			
	5y	**-0.59**	**0.01**	-1.05	-0.12
	6y	**-0.76**	**< 0.001**	-1.23	-0.28
	7y	**-0.77**	**< 0.001**	-1.24	-0.30
PHN*child age	4y	0			
	5y	0.07	0.56	-0.16	0.30
	6y	0.23	0.06	-0.01	0.48
	7y	0.22	0.06	-0.01	0.46
Child sex	female	**-1.55**	**< 0.001**	-2.42	-0.68
	male	0			
Sum of CM forms (at least moderate)					
Intercept		**8.47**	**< 0.001**	7.78	9.16
Sum of CM experiences	cont.	**0.81**	**0.01**	0.19	1.44
Child age	4y	**0**			
	5y	**-0.58**	**0.02**	-1.04	-0.11
	6y	**-0.73**	**< 0.001**	-1.22	-0.24
	7y	**-0.73**	**< 0.001**	-1.20	-0.25
Sum of CM*child age	4y	0			
	5y	0.06	0.84	-0.41	0.62
	6y	0.18	0.56	-0.26	0.76
	7y	0.28	0.33	-0.18	0.86
Child sex	female	**-1.52**	**< 0.001**	-2.38	-0.66
	male	0			

CL: Confidence limit, CM: childhood maltreatment, cont.: continuously measured, SDQ: Child behaviour difficulties; y: years, p values <0.05 are written in bold

## Discussion

The present study is the first to assess the association of maternal CM exposure with trajectories of child`s mental health measures in a prospective birth cohort. Our results add to the existing literature by a detailed trajectorial description on an annual base and a two-dimensional operationalization of maternal mental health burden by introducing maternal duration and strength of anxiety/depression symptoms in our models. The findings indicate that maternal CM is associated with more behavioural and emotional difficulties in children. While behavioural and emotional difficulties decreased in children of mothers without CM experience between the age of 4 and 7, this decrease tended to be mitigated in children of mothers with CM experience. This was mediated by maternal symptoms of anxiety and depression. Male sex of the offspring was not only associated with more behavioural and emotional difficulties but also with higher maternal symptoms of anxiety and depression.

### Maternal CM and child mental health

An association between maternal CM and child mental health was often shown [9, 10]. We add to this literature by focusing on trajectories of behavioural and emotional difficulties in the offspring. Overall behavioural and emotional difficulties decreased between the age of 4 to 7 years in boys and girls, an observation in line with findings from representative samples [20]. However, even being not consistently statistically significant in mixed model analyses, the difference between behavioural and emotional difficulties in children of mothers with and without CM experiences showed a trend to increase with older age of the children. Significant differences in child SDQ for maternal emotional neglect and the sum of CM forms experienced by the mother at her child’s age 7 were shown. This finding is supported by a study based on prospective data of Finnish adolescents with increased differences in the risk for incident behavioural and emotional difficulties in dependence of maternal adversities with increasing age [31]. In an Australian cohort, the effect of maternal mental health on child mental health increased over time [14]. In contrast to these results assessed on an annual base, other literature does not reveal a moderating effect of child age. One reason may be the categorization of age into groups above and beyond the age of 8 years [9].

Interestingly, descriptive analyses found significant differences in behavioural and emotional difficulties in the offspring for maternal emotional neglect and the sum of experienced forms of CM. Similarly, in mixed models, besides the sum of experienced forms of CM, maternal emotional abuse and emotional neglect significantly predicted behavioural and emotional difficulties in the offspring. The role of emotional abuse and neglect on the development of children is often overlooked in clinics and research. However, research suggests even a higher effect of emotional compared to physical and sexual abuse [27]. Emotional abuse and neglect are known to be stronger associated with anxiety and depression disorders compared to sexual abuse [26], an important mediator of CM and mental health in the offspring in our study. Maternal emotional abuse and neglect significantly affects parenting, being e.g. associated with lower levels of parental self-efficacy and maternal punitiveness and subsequent maternal-child interaction [35], and thus being critically for the mental health of children.

Our results point towards an intergenerational effect of emotional abuse and neglect and underline the major importance of non-physical CM forms. However, although relative values are largely comparable to the general population [36], the total number of mothers who have experienced the different forms of CM in our sample was quite small.

The risk of individuals who have experienced CM to develop a psychiatric disorder is on average at least twice as high as the risk of non-exposed individuals [2, 37]. The effect we see on mental health of children is significantly lower which is in line with the literature [9], although longer follow-ups until adulthood are needed to ensure comparability. However, our data indicate that the effect of maternal CM is intergenerational.

### The role of maternal mental health

As hypothesized, maternal anxiety and depression significantly mediated the association between maternal emotional abuse and neglect and child behavioural and emotional difficulties which is in line with the literature [12]. Surprisingly, we found no significant association between maternal physical and sexual abuse and maternal symptoms of anxiety/depression which is in contrast to previous research [1, 2, 38]. This may be due to our relatively small sample size and the low proportion of mothers who experienced sexual abuse.

Importantly, in our approach, symptoms of anxiety and depression were operationalized as “area under the curve”, thereby showing maternal internalizing problems during time course and not at one single time point. This is novel, taking into account not only severity but also chronicity of these symptoms and adds to the existing literature by combining these dimensions. Results indicate a dose-response relationship between the burden of maternal symptoms of anxiety and depression and child mental health. This shows the relevance of interventions at any time point, also beyond infancy and toddler age. Maternal mental illness negatively affects child mental health, but effective treatment of maternal depression is associated with a significant decrease in child psychiatric symptoms [13].

Focusing on a developmental perspective, experiences such as CM can influence manifold systems over time, resulting in processes that impact e.g. family systems in a cumulative cascade across systems and generations. Thus, parental CM and consequent mental health problems can result in multifaceted ways accumulating over time [21], potentially generating maladaptation limiting the healthy development [39]. Hence, the effect of maternal mental health problems may be cumulative, influencing the mental health of the offspring in a cascadian way. However, maternal mental health is only one route via which maternal CM may affect the mental health of the offspring. Possible other reasons for this intergenerational transmission comprise both, nature and nurture aspects at different development stages. Prenatally, changes in maternal factors in the oocyte or during pregnancy may impact child mental health [11]. Other postnatal CM-associated maternal factors are an enhanced risk for higher maternal stress, harsh parenting or perpetration of CM [10, 40]. Of note, these factors are strongly interrelated, as e.g., maternal mental illness is linked to higher maternal stress, and a higher risk of harsh parenting and perpetration of CM [41]. Further prospective studies across generations that comprise somatic and mental health are needed to disentangle this highly complex interplay of genetics, stress biology, mental health and parenting.

### Moderating role of child sex

Interestingly, male sex of the child was associated with increased symptoms of anxiety and depression in mothers. Literature shows heterogeneous results for the interplay between maternal and child mental health and child sex [42, 43]. However, when maternal CM is taken into account, a greater vulnerability for boys was shown before for the indirect effects of maternal CM via anxiety and depression [18]. Our results point in the same direction. In our findings, older age and female sex were associated with lower behavioural and emotional difficulties, which is in line with the results of representative samples [20]. Sex is known to significantly affect the development of psychopathology, as there are relevant gender differences regarding mental health problems [44]. Child sex is further suggested to moderate the effect of maternal and child mental health [42].

### Limitations

A central limitation of the present study is that CM experience and maternal and child mental health are based on maternal self-report. Maternal rating of the child mental health might have been affected by CM experience and mental health with maternal depression resulting in overreporting of child mental health problems [45]. The time of the CM assessment might further be considered critical as a decline in recall accuracy was proposed suggesting that an earlier assessment might result in less memory bias [36]. However, underreports tend to be more frequent than overreports, i.e. those who were subject to abuse often provided false negative reports [46], which may result into lower observed than true effects. A lack of distance to the experiences at a very young age might also be an issue [47].

Besides, as recruitment took place only at one site and in a relatively affluent city in the South of Germany, and selection bias due to stronger loss to follow-up of non-German families or families with a lower socio-economic status cannot be excluded (Online Resource Table 1), our sample cannot be considered as representative of the German population. However, behavioural and emotional difficulties in our sample were comparable but slightly lower compared to those in the German Health Interview and Examination Survey for Children and Adolescents (KiGGS), a nationally representative survey including 14,835 children aged 3–17 years [20].

Rates of emotional abuse were slightly higher in our sample compared to a sample of the general population in Germany, while all other forms were lower, with significantly lower rates for physical neglect in our sample [36]. For the treatment of missing values we used slightly different approaches for dependent and independent variables and covariates. Nevertheless, we believe that these different approaches do not influence the results due to the small numbers of missing values (see previous paragraphs). Covariates were selected based on previous reports and based on availability of the data. Further covariates like maternal social support or family income might also be of interest, however, have not been assessed in our study. A possible underreporting of maternal mental health problems by focusing solely on maternal anxiety and depression symptoms has to be taken into account.

## Conclusion

Overall, despite these limitations, the presented data provide an important first insight into the relevance of maternal CM on the trajectories of mental health in the offspring and highlight the importance of chronicity and severity of minor symptoms of maternal anxiety and depression in this interplay. Based on a somatically and psychologically well characterized prospective cohort, our study underlines the high complexity of the development of mental health problems in childhood and the necessity for comprehensive approaches to better understand the relationship between maternal CM, maternal mental health and child mental health. Furthermore, our results reveal a greater vulnerability of male gender in this interplay.

In sum, our results indicate that the association between maternal CM and child behavioural and emotional difficulties increases with older child age. Thus, further research with longer follow-ups up into adolescence and adulthood is needed. The here shown cumulative effect on symptoms of anxiety and depression on child mental health underlines the relevance of effective treatment for mentally ill parents. Focusing the developmental cascade of consequences of maternal CM shown here, early intervention and support for children is crucial. The intergenerational consequences of CM are underlined by our study, again emphasizing the need for efficient child protection systems. Taking into account the high economic costs of mental health problems and the intergenerational sequelae of CM on mental health, child protection measures seem to be effective not only from an individual but also from a societal, economical dimension. People working in the mental health area, such as psychiatry and child and adolescent psychiatrists must be aware of child protection issues in particular, treating a high risk group of parents and children, and of interventions to support their parents and families.

## Electronic supplementary material

Below is the link to the electronic supplementary material.

Supplementary Material 1

## Data Availability

Availability of data and material (data transparency): Obtaining data and code is possible through personal contact with the corresponding author (stefanie.braig@uni-ulm.de).
