# Supplementary material for: Maternal child maltreatment and trajectories of offspring behavioural and emotional difficulties from age 4 to 7 years – results from a prospective birth cohort study
Source: Eur Child Adolesc Psychiatry. 2024 Jul 22;34(3):1039–50. doi: 10.1007/s00787-024-02534-3 (PMC11909082; doi:10.1007/s00787-024-02534-3)
Supplement: Supplementary file 1 — Supplementary Material 1 [file 787_2024_2534_MOESM1_ESM.pdf]

Supplement

Vera Clemens, Deborah Wernecke, Jörg M. Fegert, Jon Genuneit, Dietrich Rothenbacher, Stefanie Braig: *Maternal child maltreatment and trajectories of offspring behavioural and emotional difficulties from age 4 to 7 years – results from a prospective birth cohort study;*
European Child and Adolescent Psychiatry;
Corresponding author: Stefanie Braig, Ulm University, stefanie.braig@uni-ulm.de

Fig. 1 Mean predicted maternal anxiety/depression symptoms and 95% confidence intervals according to maternal CM (adjusted for child sex)

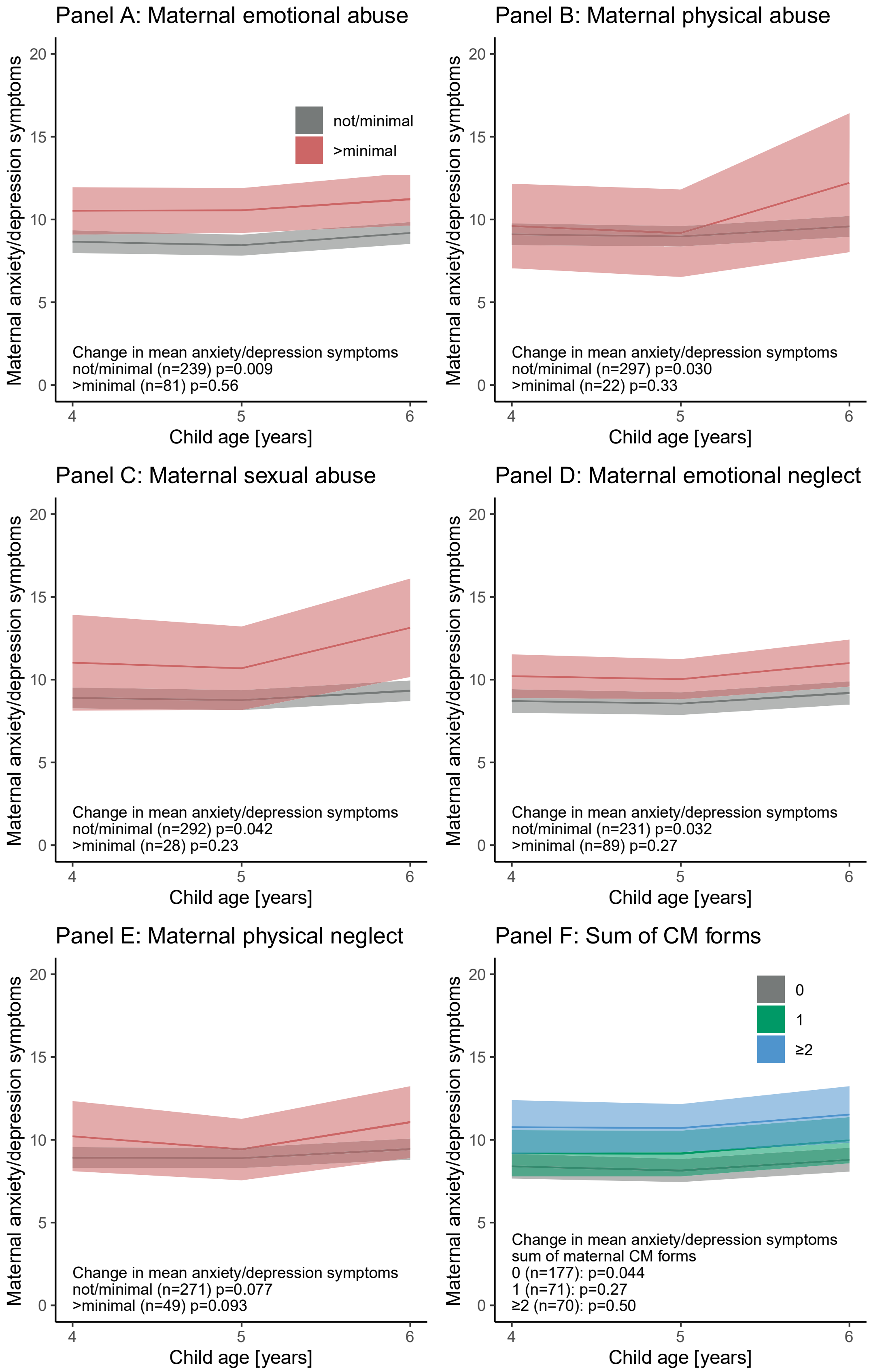

CM: childhood maltreatment

Fig. 2 Path analysis

Panel A: Maternal emotional abuse (AIC: 6554.10, BIC: 6591.91)

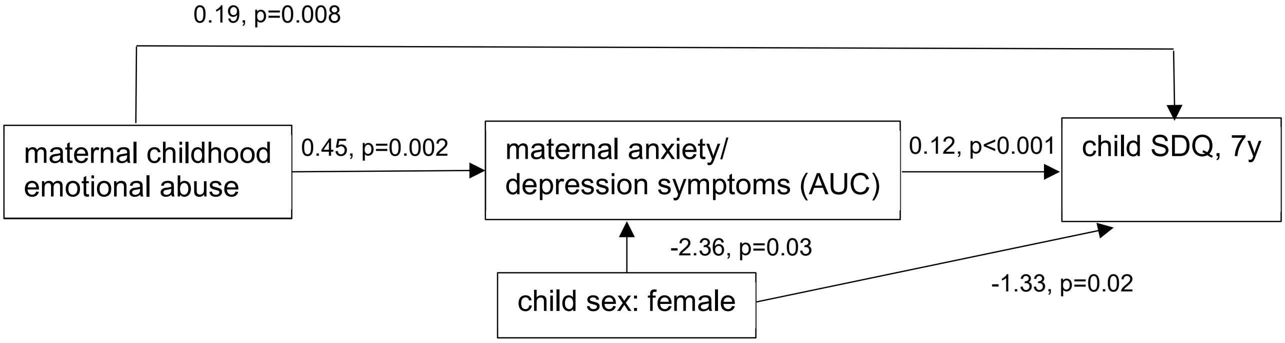

Panel B: Maternal physical abuse (AIC: 6105.10, BIC: 6142.87)

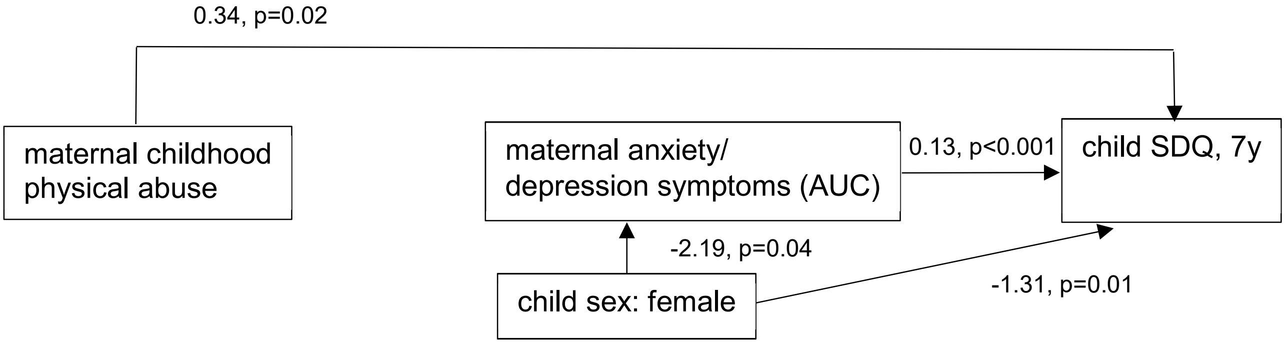

Panel C: Maternal sexual abuse (AIC: 6184.78, BIC: 6222.59)

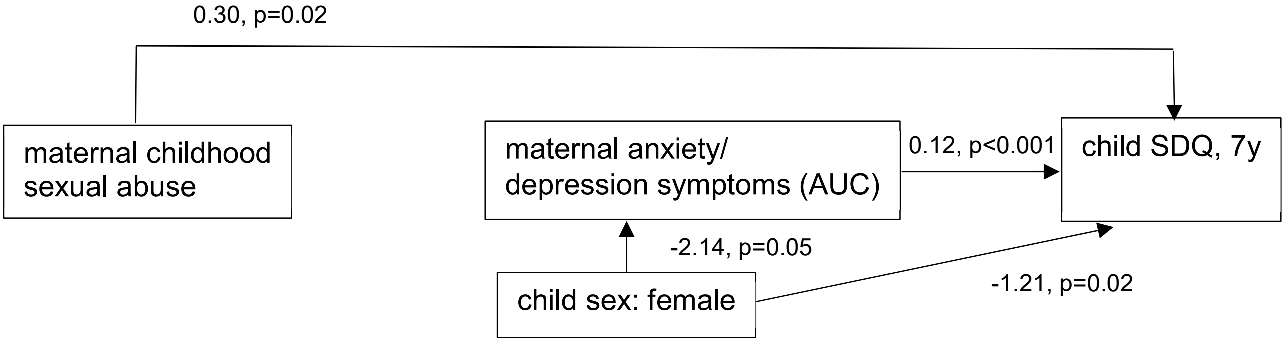

Panel D: Maternal emotional neglect (AIC: 6371.26, BIC: 6409.07)

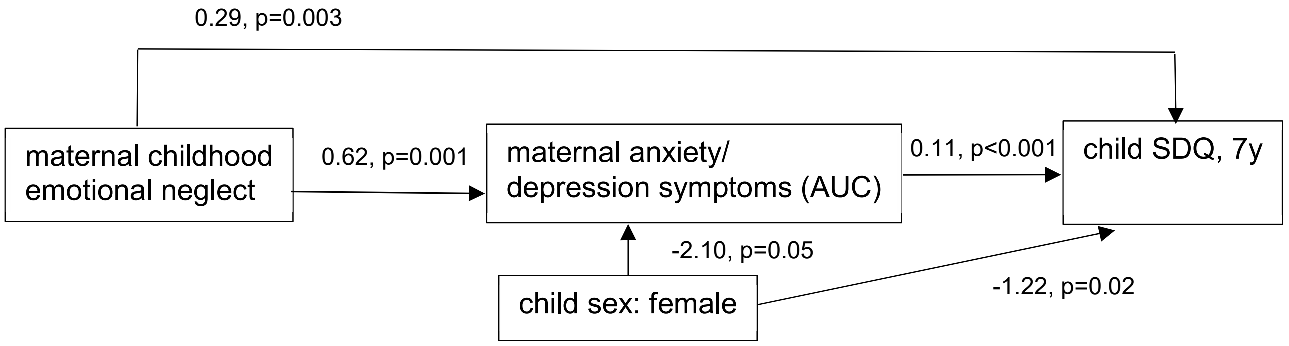

Panel E: Maternal physical neglect (AIC: 6155.79, BIC: 6193.60)

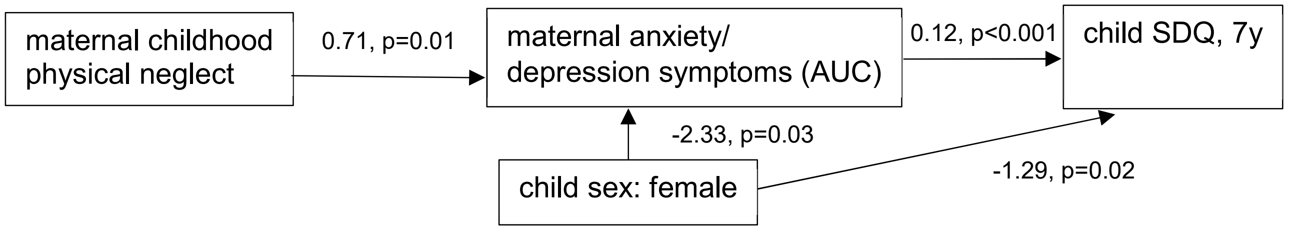

paths with p≥0.05 are omitted in the figure, AIC: Akaike information criterion, AUC: Area under the curve, BIC: Bayesian information criterion, y: years

Table 1: Comparison between study population and baseline population

	**Study population (n=327)**	**Baseline population (n=934)**	
	**(%)**	**n (%)**	**95% Confidence Interval**
Maternal age at childbirth [years]			
≤25	**3.1%**	60/933 (6.4%)	4.9%; 8.2%
26-35	70.3%	642/933 (68.8%)	65.7%; 71.8%
≥36	26.6%	231/933 (24.8%)	22.0%; 27.7%
Mean (SD)	**33.5 (4.5)**	32.7 (4.8)	32.4; 33.0
Maternal nationality German	**93.9%**	823/926 (88.9%)	86.7%; 90.8%
Maternal education (<12 years)	**28.0%**	370/915 (40.4%)	37.2%; 43.7%
Single motherhood at childbirth	**1.2%**	25/921 (2.7%)	1.7%; 3.8%
Maternal smoking before pregnancy	**17.8%**	29/155 (18.7%)	24.6%; 30.4%

bold letters indicate statistically significant associations, i.e. proportions not within the 95% confidence interval
